# Fermentation by *Wickerhamomyces anomalus* Improved Production Yield of Fructooligosaccharides Through Transglycosidation of β-Fructofuranosidase

**DOI:** 10.3390/foods15030592

**Published:** 2026-02-06

**Authors:** Hong Liu, Qiaojuan Yan, Susu Han, Xiaoxiao Wang, Yanxiao Li, Zhengqiang Jiang

**Affiliations:** 1 Key Laboratory of Food Bioengineering (China National Light Industry), College of Food Science & Nutritional Engineering, China Agricultural University, Beijing 100083, China; 2 College of Engineering, China Agricultural University, Beijing 100083, China

**Keywords:** fructooligosaccharides, *Wickerhamomyces anomalus*, β-fructofuranosidase, FOS purification, sequential action

## Abstract

Fructooligosaccharides (FOS), an important prebiotic, are widely used in the food industry. β-Fructofuranosidases are commonly used for FOS production with yield of 55~60% (crude FOS syrup). The by-products glucose and fructose are produced during enzymatic conversion of FOS. Thus, the strategy for FOS production with high content (higher than 95%) has long been a topic of concern. In this study, a novel yeast *Wickerhamomyces anomalus* CAU331 was isolated from *Daqu* and applied for fermentation of crude FOS syrup. Impurities of glucose and fructose in the syrup were metabolized, which yielded a FOS content of 87.6%. Subsequently, the β-fructofuranosidase (AnFTase70) was added and synergistically worked with *W. anomalus* CAU331. A maximum FOS content of 95.1% with a concentration of 288.1 g/L and productivity of 6.26 g/L/h was obtained through the sequential action of β-fructofuranosidase and *W. anomalus* CAU331 in a 200 L fermenter. Moreover, the FOS components were composed of 19.2 g/L 1-kestose (GF_2_), 127.8 g/L nystose (GF_3_), 115.8 g/L 1F-fructofuranosylnystose (GF_4_), and 25.5 g/L kestohexaose (GF_5_). The findings gained in this study might provide a cost-effective approach for the production of FOS with high purity and expand their applications as functional food materials.

## 1. Introduction

Fructooligosaccharides (FOS) are linear hetero-oligosaccharides composed mainly of 1-3 fructosyl units linked by β-2,1 glucosidic bonds and represent an important prebiotic widely used in the food industry [1]. Commercial FOS typically consist of 1-kestose (GF_2_), nystose (GF_3_), and 1F-fructofuranosylnystose (GF_4_) [2], while higher degrees of polymerization (DP) like kestohexaose (GF_5_) and 1-kestoheptaose (GF_6_) are also recognized [3]. These oligosaccharides selectively stimulate the growth of beneficial intestinal bacteria, such as *Bifidobacteria* sp. and *Lactobacillus* sp, which ferment FOS into short-chain fatty acids in the colon, thereby supporting immune function [4,5]. Additionally, FOS also offer benefits like low caloric value, blood lipid reduction, and enhanced absorption of trace elements [6]. As a result, FOS are commonly employed as sugar substitutes and functional ingredients to mitigate risks of obesity, diabetes, inflammation, and dental caries [7,8]. FOS with higher DP exhibit stronger distal colon prebiotic effects due to their lower susceptibility to rapid fermentation in the proximal colon [9]. Hence, high-DP FOS are of significant value for practical applications. Currently, FOS are mainly produced commercially through enzymatic conversion of sucrose [10]. However, this process still faces limitations such as low FOS purity and concentration, low product DP, and poor stability. A large amount of glucose is generated as a by-product, resulting in a sucrose conversion yield of only 0.55~0.60 g_FOS_/g_sucrose_, which restricts large-scale applications of FOS [11,12]. Therefore, it is crucial to establish an industrially viable and efficient production process for high-content FOS.

The production of high-content FOS requires the combination of two strategies: enzymatic conversion and the removal of monosaccharides [13,14]. Enzymatic conversion by β-fructofuranosidases from *Aspergillus* sp., *Aureobasidium* sp., and *Penicillium* sp. primarily acts to transform sucrose into crude FOS syrup [15,16], while the latter employs methods such as membrane techniques, chromatographic methods, enzyme treatment, and microbial fermentation to remove monosaccharides [17,18]. Conventional FOS purification methods, such as membrane filtration and chromatography, often require repeated operations and lead to significant FOS loss alongside monosaccharides removal [19]. Similarly, enzymatic removal of glucose faces limitations, including incomplete precipitation and the need for additional purification steps [20]. In contrast, microbial fermentation offers a distinct advantage by directly consuming the by-product glucose during the enzymatic catalysis process, thereby enhancing both conversion efficiency and product purity in a more integrated manner [4]. Recent studies have advanced high-content FOS production through integrated microbial and enzymatic strategies, achieving FOS content of 81.6–96.6% and concentrations up to 195.9 g/L [17,21,22,23]. Nevertheless, broader application is limited by persistently low FOS yields and high operational cost, primarily due to inefficient microbial sugar consumption. Thus, future efforts should focus on the development of more efficient sugar-consuming strains and optimized purification processes to overcome these key industrial bottlenecks.

In our previous study, a β-fructofuranosidase (AnFTase70) from *Aspergillus niger* was produced, which has high FOS synthesis ability [12]. Here, a strain of *Wickerhamomyces anomalus* CAU331, exhibiting high glucose consumption capacity and remarkable sugar tolerance, was isolated from *Daqu*. It was deposited in the China General Microbiological Culture Collection Center under accession number CGMCC 23766 and was applied to improve the purity of FOS. Furthermore, purification strategies were developed for the production of high-content FOS syrup.

## 2. Materials and Methods

### 2.1. Experimental Materials

FOS standards of 1-kestose (GF_2_), nystose (GF_3_), 1F-fructofuranosylnystose (GF_4_), and kestohexaose (GF_5_) were obtained from Shanghai yuanye Bio-Technology Co., Ltd. (Shanghai, China). Fructose, glucose, and sucrose standards were purchased from Sigma (Saint Louis, MO, USA). The β-fructofuranosidase (AnFTase70) was prepared according to the method that was previously described by Han et al. [12]. Briefly, the enzyme expressed in *A. niger* FBL-B was fermented in a 200 L fermenter under 450 rpm agitation and 0.5 vvm airflow at 34 °C for 168 h. The β-fructofuranosidase activity of 15,006 U/mL was produced with a protein concentration of 23.2 g/L.

### 2.2. Isolation and Identification of Yeast for Purification Crude FOS Syrup

*Daqu* samples collected from Luohe (Henan, China), were serially diluted from to 10^−1^ to 10^−6^ and spread onto YEPD agar plates containing yeast extract (10 g/L), tryptone (20 g/L), glucose (20 g/L), and agar (20 g/L). After incubation at 30 °C for 48 h, rapidly growing yeast-like colonies were selected and purified through repeated streaking on fresh YEPD agar. Subsequently, purified isolates were inoculated into YEPD seed medium (yeast extract 10 g/L, tryptone 20 g/L and glucose 20 g/L) and cultivated at 30 °C for 24 h. The strains were then assessed for their ability to metabolize the glucose present in crude FOS syrup, which was prepared following the procedure previously described by Han et al. [12]. Briefly, β-fructofuranosidase (5 U/mL) was added to a 250 mL shake flask containing 50 mL of 300 g/L sucrose solution and was incubated at 45 °C for 4 h. The resulting crude FOS syrup was then heated at 100 °C for 5 min to inactivate the enzyme. Each seed culture was inoculated at 10% (*v*/*v*) inoculum size into the crude FOS syrup and fermented at 30 °C for 30 h while shaking at 200 rpm. One isolate exhibiting high glucose consumption efficiency was designated as CAU331, preserved in 40% (*v*/*v*) glycerol at −80 °C, and deposited in CGMCC (No. 34766).

Genomic DNA was extracted from pure cultures using a commercial yeast DNA kit (Tiangen Biotechnology Co. Ltd., Beijing, China). The 18S rDNA region was amplified, sequenced and analyzed for species identification. A phylogenetic tree was constructed with the neighbor-joining method in MEGA7.0. Colony morphology was observed on YEPD agar, while cellular morphology was examined under an optical microscope.

### 2.3. Preparation of Yeast Direct-Vat Set Starter and Fermentation Optimization for Crude FOS Syrup

*Wickerhamomyces anomalus* CAU331 was reactivated on YEPD agar plates and incubated at 30 °C for 48 h. Colonies were inoculated into YEPD liquid medium and grown for 20 h at 30 °C with agitation at 200 rpm. The resulting seed culture (10%, *v*/*v*) was transferred into a 5 L fermenter containing 3 L of YEPD medium. Fermentation was carried out at 30 °C with continuous stirring at 200 rpm and an aeration rate of 1.0 vvm for 48 h. The pH was automatically maintained at pH 6.0 by the addition of NH_4_OH or H_3_PO_4_. A feed medium containing yeast extract (100 g/L), tryptone (200 g/L), and glucose (200 g/L) was supplied whenever the residual glucose concentration fell below 5 g/L. After fermentation, cells were harvested by centrifugation at 4000 rpm for 10 min to prepare the direct-vat set starter.

Fermentation conditions were initially optimized in shake flasks using a one-factor-at-a-time approach. The conditions included tryptone concentration (10 g/L, 15 g/L, 20 g/L, and 25 g/L), initial pH (5.5, 6.0, 6.5, and 7.0), inoculum size (0.05%, 0.1%, 0.3%, and 0.5%), temperature (25 °C, 30 °C, 35 °C, and 40 °C) and fermentation time (12 h, 18 h, 24 h, and 30 h). Subsequently, response surface methodology (RSM) with a Box–Behnken design (BBD) was applied to optimize three key factors, each tested at three levels, resulting in seventeen experimental runs (Table S1). Data were analyzed by analysis of variance (ANOVA) using Design-Expert 8.0.6 to fit a quadratic model and identify optimal conditions, which were then adopted for further studies.

### 2.4. Preparation Optimization for Crude FOS Syrup

To optimize enzyme dosage for crude FOS syrup preparation, different concentrations of β-fructofuranosidase (1, 3, 5, and 10 U/mL) were added to a 250 mL shake flask containing 50 mL of 500 g/L sucrose solution, followed by incubation at 45 °C for 8 h. In parallel, sucrose concentrations (300, 500, 600, and 700 g/L) were also optimized using a fixed enzyme dosage of 5 U/mL.

FOS content and yield were calculated according to the equations presented below. FOS content(%) = (GF_2_ + GF_3_ + GF_4_ + GF_5_)/(fructose + glucose + sucrose + GF_2_ + GF_3_ + GF_4_ + GF_5_) × 100%; FOS yield(%) = (GF_2_ + GF_3_ + GF_4_ + GF_5_)/initial sucrose × 100%.

### 2.5. Production Strategy of High-Content FOS Syrup

A 5 U/mL dosage of β-fructofuranosidase was added to a 200 L fermenter (Bailun, Shanghai, China) containing 120 L of 500 g/L sucrose solution, and the reaction was carried out at 45 °C with agitation at 100 rpm for 4 h. The crude FOS syrup was then heated at 100 °C for 5 min to inactivate the enzyme. Meanwhile, the yeast direct-vat set starter was inoculated at 0.3% (*w*/*v*) into the fermenter. Fermentation proceeded at 30 °C with stirring at 400 rpm and an aeration rate of 0.5 vvm for 34 h. An additional 5 U/mL of β-fructofuranosidase was subsequently introduced, and fermentation continued for another 10 h, during which the pH was maintained at pH 6.0 by automatic addition of NH_4_OH or H_3_PO_4_. Samples were periodically collected throughout the process for analysis of cell biomass and sugar concentration.

### 2.6. Determination of Cell Biomass and Sugars

Cell biomass was determined by wet cell weight. An amount of 1 mL of the fermentation broth was centrifuged at 10,000 rpm for 10 min, and the pellet was washed twice with distilled water. Sugar composition in the FOS syrup was analyzed using an Agilent 1260 HPLC system (Agilent Technologies, Santa Clara, CA, USA) equipped with a refractive index detector and a XBridge^R^ BEH Amide column (4.6 × 250 mm, Waters, Rydalmere, Australia). The mobile phase was acetonitrile/water (72:28, *v*/*v*) at a flow rate of 0.6 mL/min at 45 °C [12].

### 2.7. Statistical Analysis

Results are shown as the mean ± standard deviation of triplicate experiments. Data were analyzed with SPSS 16.0 and Origin 9.2, and differences were considered significant at *p* < 0.05.

## 3. Results and Discussion

### 3.1. Isolation and Identification of Yeast for Purification of Crude FOS Syrup

**Figure 1:**
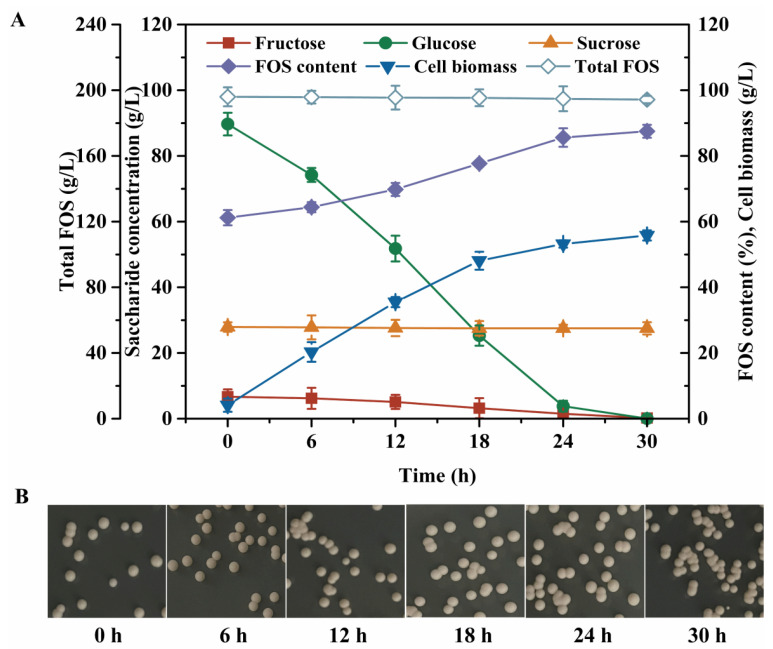
Time course of crude FOS syrup fermented by strain CAU331. Concentrations of fructose, glucose, sucrose, FOS content, total FOS and cell biomass (**A**). Colony morphology observed on YEPD agar plates (**B**). Symbols denote: fructose concentration (■), glucose concentration (●), sucrose concentration (▲), FOS content (◆), cell biomass (▼) and total FOS (◇). β-Fructofuranosidase (5 U/mL) was added to a 250 mL shake flask containing 50 mL of 300 g/L sucrose solution and incubated at 45 °C for 4 h. The crude FOS syrup was heated at 100 °C for 5 min to inactivate the enzyme. Subsequently, a seed culture of strain CAU331 was inoculated at 10% (*v*/*v*) inoculum size and fermented at 30 °C for 30 h with shaking at 200 rpm. All experiments were conducted in triplicate, and error bars indicate standard deviations.

**Figure 2:**
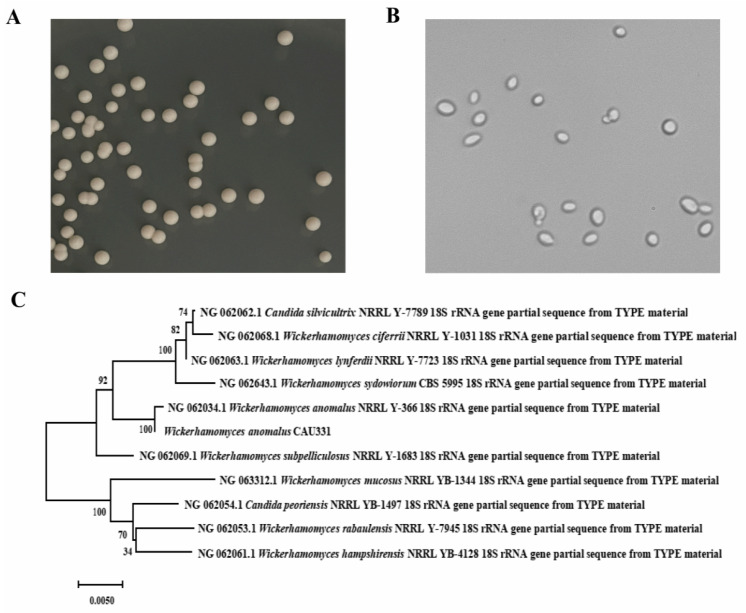
Morphological and phylogenetic characterization of *W. anomalus* CAU331. Colony morphology observed on YEPD agar plates (**A**). Cellular morphology observed under an optical microscope (400× magnification) (**B**). Phylogenetic tree constructed using the neighbor-joining method in MEGA7.0 with 1000 bootstrap replicates (**C**).

As shown in Figure 1A, the strain CAU331 exhibited rapid growth during the first 24 h, achieving a cell biomass of 53.2 g/L. Concurrently, the glucose concentration sharply declined from 89.7 g/L to 3.8 g/L, while fructose concentration decreased from 6.7 g/L to 1.5 g/L. The FOS content increased from 61.2% to 85.6%, whereas sucrose concentration remained constant, ending at 27.5 g/L. Throughout the process, total FOS concentration showed negligible change, and the colonial morphology of strain CAU331 was unchanged (Figure 1B). Based on its efficient glucose removal and stable FOS retention, the strain CAU331 was selected for further purification of crude FOS syrup. Colony morphology analysis revealed that CAU331 forms circular, milky-white, raised colonies with smooth surfaces and edges (Figure 2A). Microscopic observation indicated oval-shaped and budding spores (Figure 2B). Phylogenetic identification based on the 1667 bp 18S rDNA sequence showed 100% identity with *Wickerhamomyces anomalus* NRRL Y-366 (NG062034.1) (Figure 2C), confirming the strain as *Wickerhamomyces anomalus.*

Microbial fermentation has emerged as a promising strategy for the removal of low-molecular-weight saccharide by-products from enzymatically produced FOSs [23]. Several microbial strains, such as *S. cerevisiae* 11,982 [21], *W. anomalus* GXL-22 [17], and *B. coagulans* [19], have been effectively applied to eliminate mono- and disaccharides from crude FOS mixtures. Specifically, *S. cerevisiae* 11982 preferentially metabolized glucose over fructose, reducing its concentration from 54.7 g/L to 2.4 g/L at a rate of 1.09 g/L/h [21]. Similarly, *W. anomalus* GXL-22 demonstrated efficient removal of glucose, fructose, and sucrose from crude FOS syrup (total sugar 300 g/L), with glucose declining from 72.6 g/L to 3.2 g/L at 3.47 g/L/h [17]. In contrast, *B. coagulans* showed a lower glucose consumption rate (<1.0 g/L/h) and was inhibited at high total sugar concentrations (150 g/L), likely due to high osmotic pressure [19,24]. In the present study, *W. anomalus* CAU331 displayed high glucose and fructose metabolic capability, with a glucose consumption rate of 3.58 g/L/h and 4.12 g/L/h in crude FOS syrup containing 300 g/L and 500 g/L total sugars, respectively. Fructose utilization proceeded more slowly, consistent with the glycotropic characteristic reported for this yeast genus, wherein fructose metabolism is initiated only after glucose depletion [22]. This differential sugar uptake aligns with earlier observations and supports the strain’s suitability for FOS purification [21]. Owing to its high glucose consumption capacity and remarkable sugar tolerance, *W. anomalus* CAU331 represents an excellent candidate for the purification of crude FOS syrup.

### 3.2. Optimization of Fermentation Conditions for Purification of Crude FOS Syrup by Direct-Vat Set Starter

**Figure 3:**
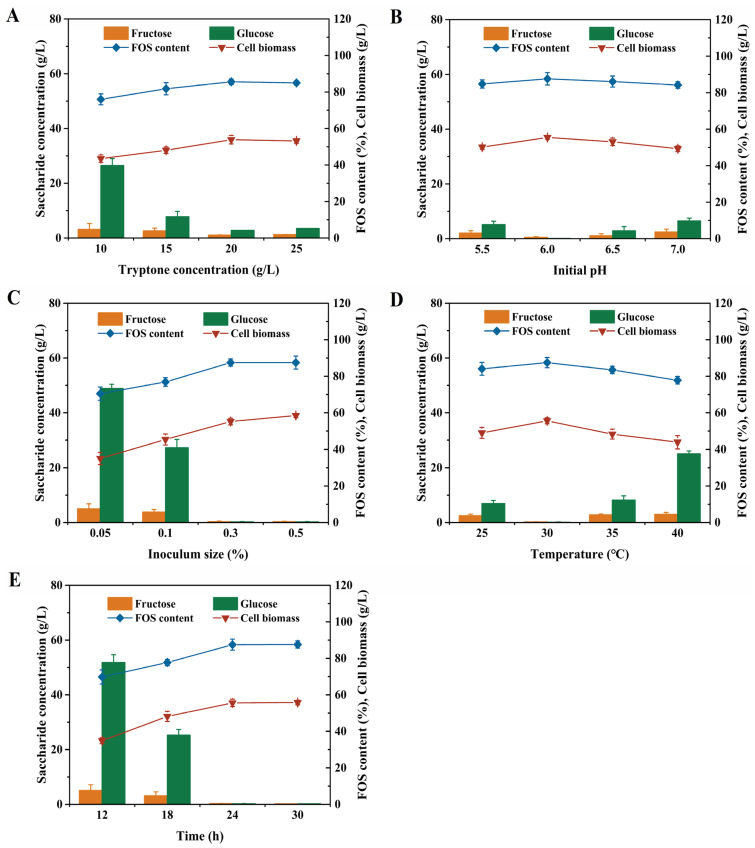
Optimization of fermentation conditions for purification of crude FOS syrup by *W. anomalus* CAU331. Effects of tryptone concentration on crude FOS syrup purification (**A**). Effects of initial pH on crude FOS syrup purification (**B**). Effects of inoculum size on crude FOS syrup purification (**C**). Effects of fermentation temperature on crude FOS syrup purification (**D**). Effects of fermentation time on crude FOS syrup purification (**E**). Crude FOS syrup was prepared by incubating 50 mL of 300 g/L sucrose solution with 5 U/mL β-fructofuranosidase in a 250 mL shake flask at 45 °C for 4 h, followed by heat inactivation at 100 °C for 5 min. Subsequently, the seed culture of *W. anomalus* CAU331 was inoculated into the crude FOS syrup and fermented at 200 rpm for 30 h. All experiments were conducted in triplicate, and error bars indicate standard deviations.

To enhance the growth and saccharide consumption rate of *W. anomalus* CAU331, fermentation conditions were optimized through a single-factor experiment approach (Figure 3). In Figure 3A, the impact of tryptone concentration on saccharide consumption capacity is depicted, with 20 g/L tryptone resulting in FOS content of 85.6%. As shown in Figure 3B,D, saccharide consumption gradually declined at temperatures more than 35 °C. However, over a pH range of pH 5.5~7.0 and a temperature range of 25 °C~35 °C, *W. anomalus* CAU331 maintained stable saccharide consumption, with FOS content consistently above 84%. Thus, pH 6.0 and 30 °C were selected for further experiments. Inoculum size directly affected the lag period and saccharide consumption rate, FOS content initially rose and then stabilized with increasing inoculum levels (Figure 3C). At 0.3% inoculum size, FOS content peaked at 87.5%, and fructose and glucose were nearly depleted, establishing this as the optimal inoculum size. Based on these results, the time course for the purification of crude FOS syrup by *W. anomalus* CAU331 was further examined (Figure 3E). During the first 24 h, FOS content increased significantly while residual saccharides declined markedly, reaching a maximum FOS content of 87.5% with residual fructose and glucose concentrations of 0.3 g/L and 0.2 g/L, respectively. Both FOS content and residual saccharide concentrations remained stable within 24 h. The fermentation conditions were thus optimized as follows: tryptone concentration 20 g/L, initial pH 6.0, inoculum size 0.3%, fermentation temperature 30 °C, and fermentation time 24 h. Subsequently, tryptone concentration (A), initial pH (B) and inoculum size (C) were further optimized using RSM for crude FOS syrup purification by *W. anomalus* CAU331. Analysis of variance (ANOVA) confirmed the high significance of the model (*p* < 0.0001, Table S2). Significant model terms included A, B, C, AC, A^2^, B^2^, and C^2^. The relationship among these variables is described by the following second-order polynomial equation:

Y = 87.38 + 1.35 × A − 0.81 × B + 9.34 × C + 0.075 × A × B − 1.38 × A × C + 0.50 × B × C − 1.67 × A^2^ − 3.84 × B^2^ − 9.54 × C^2^

**Figure 4:**
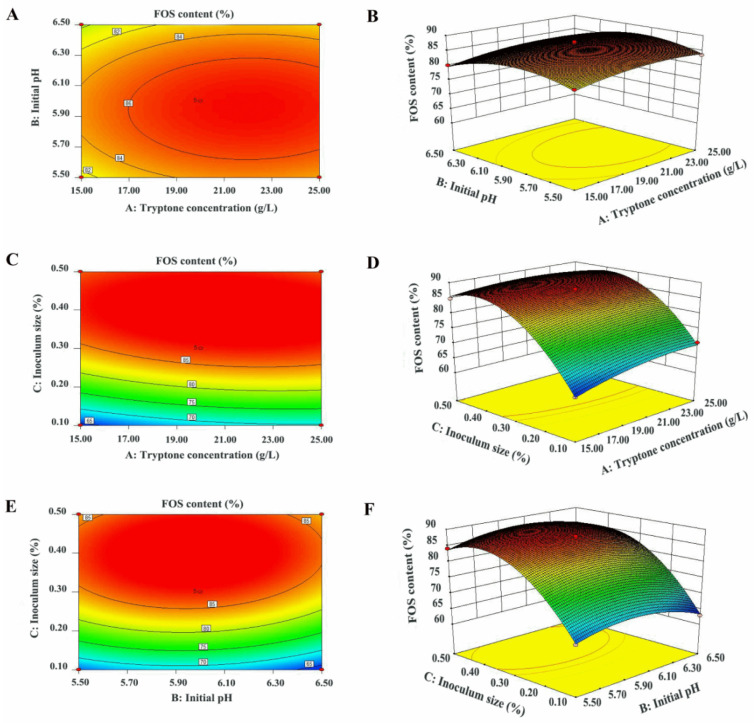
Contour and response surface plots for the purification of crude FOS syrup by *W. anomalus* CAU331. Tryptone concentration and initial pH (**A**,**B**); tryptone concentration and inoculum size (**C**,**D**); initial pH and inoculum size (**E**,**F**). The varying colors correspond to the value of the response variable, with warmer colors (like red) representing higher values and cooler colors (like blue) representing lower values. Crude FOS syrup was prepared by incubating 50 mL of 300 g/L sucrose solution with 5 U/mL β-fructofuranosidase in a 250 mL shake flask at 45 °C for 4 h, followed by heat inactivation at 100 °C for 5 min. Subsequently, the seed culture of *W. anomalus* CAU331 was inoculated into the crude FOS syrup and fermented at 200 rpm for 30 h. All experiments were conducted in triplicate, and error bars indicate standard deviations.

The interaction effects among the three variables were visualized through contour and response surface plots (Figure 4A–F). Under the optimized conditions of 21.0 g/L tryptone, initial pH 6.0, and 0.4% inoculum size, the predicted maximum FOS content was 89.7%. Validation experiments performed with these parameters achieved a maximum FOS content of 87.6% after 24 h of fermentation, which closely matched the predicted value.

To improve both the growth and saccharide consumption efficiency of the strain, various fermentation conditions—including tryptone concentration, initial pH, inoculum size, fermentation temperature, and fermentation time—are usually examined. The optimal fermentation parameters are therefore crucial for the improvement of FOS purification efficiency [17]. Nitrogen source is an indispensable nutrient for microbial metabolism. Simultaneously, efficient saccharide consumption heavily relies on nitrogen source availability [25]. The highest FOS content of 85.6% was achieved with 20 g/L tryptone, suggesting that *W. anomalus* CAU331 preferentially utilizes organic nitrogen sources during saccharide consumption. Nitrogen source selection is known to be strain-specific, with no universal option applicable to all microorganisms [26]. The optimal initial pH and temperature for *W. anomalus* CAU331 were identified as pH 6.0 and 30 °C, consistent with reports that yeast-mediated saccharide consumption typically occurs under weakly acidic conditions [4,10,17,21]. Notably, *W. anomalus* CAU331 maintained stable saccharide consumption across an initial pH range of 5.5~7.0 and a temperature range of 25 °C~35 °C, indicating broader pH and thermal adaptability compared to previously reported strains [17,19,22,27]. Such robustness is advantageous for industrial applications. Moreover, an inoculum size of 0.3% yielded a maximum FOS content of 87.6%, substantially higher than the level achieved by *W. anomalus* GXL-22 with a 20% inoculum size [17]. These optimized conditions were conducted to support robust growth of *W. anomalus* CAU331, thereby enabling more efficient and cost-effective crude FOS syrup purification.

### 3.3. Preparation Optimization for Crude FOS Syrup

**Figure 5:**
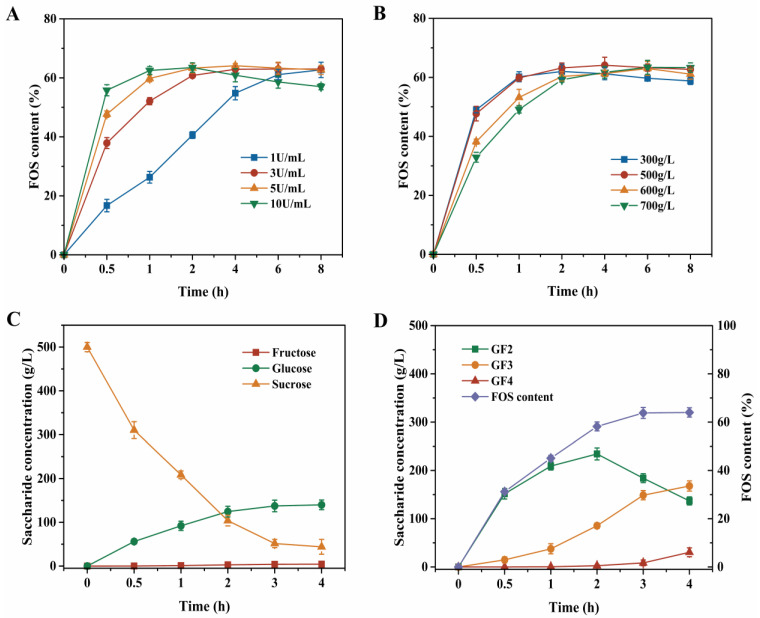
Optimization of the crude FOS syrup preparation. Effect of various β-fructofuranosidase dosage (**A**) and concentration of sucrose (**B**). Time course of crude FOS syrup preparation using 5 U/mL β-fructofuranosidase and 500 g/L sucrose solution (**C**,**D**). Fructose, glucose, and sucrose concentrations (**C**); GF_2_, GF_3_, and GF_4_ concentrations, along with FOS content (**D**). In all experiments, β-fructofuranosidase was added to 50 mL sucrose solution in a 250 mL shake flask and incubated at 45 °C. All experiments were conducted in triplicate, and error bars indicate standard deviations.

To further improve the production efficiency of FOS, the time course of crude FOS syrup preparation was studied via hydrolysis and transglycosidation under different enzyme dosages and sucrose concentrations (Figure 5). The combination of 5 U/mL β-fructofuranosidase and 500 g/L sucrose concentration was found to be optimal, yielding a maximum FOS content of 64.1% and FOS yield of 66.7% after 4 h of reaction (Figure 5A,B). Under these conditions, the syrup composition comprised 0.8% fructose (4.4 g/L), 26.7% glucose (140.0 g/L), and 8.4% sucrose (44.0 g/L) (Figure 5C). The FOS profile included 26.1% GF_2_ (136.9 g/L), 32.1% GF_3_ (167.9 g/L), and 5.8% GF_4_ (30.4 g/L) (Figure 5D).

The FOS yield achieved in the present work using β-fructofuranosidase ranks among the highest values reported in the literature for FOS production. For instance, Sangeetha et al. obtained a FOS yield of 58% in a two-stage continuous process employing *A. oryzae* CFR 202 [28]. Similarly, Dominguez et al. reported a FOS yield of 64% after 51 h in a one-step fermentation using *A. pullulans* under optimized conditions [29]. Comparable yield (64%) was also described by De la Rosa et al. with *A. oryzae* DIA-MF grown on a medium containing aguamiel and molasses [10]. In flask-scale experiments, Nobre et al. optimized temperature and initial pH for *A. ibericus* MUM 03.49, achieving a FOS yield of 53% [30]. Other whole-cell fermentation processes under optimized conditions have yielded 56% for a co-culture of *A. ibericus* MUM 03.49 and *S. cerevisiae* YIL162W [22], 55% for *P. citreonigrum* URM 4459 [31], 58% for *A. melanogenum* 33 [32], and 63% for *A. oryzae* S719 [33]. Notably, the FOS yield obtained in this study (66.7%) surpasses those reported in previous flask-level studies. Furthermore, when scaled up to a 200 L fermenter, the FOS yield increased slightly to 67.0%. These results demonstrate the great potential of the β-fructofuranosidase in this study for efficient production of high-content FOS.

### 3.4. Production of High-Content FOS by β-Fructofuranosidase and W. anomalus CAU331

**Figure 6:**
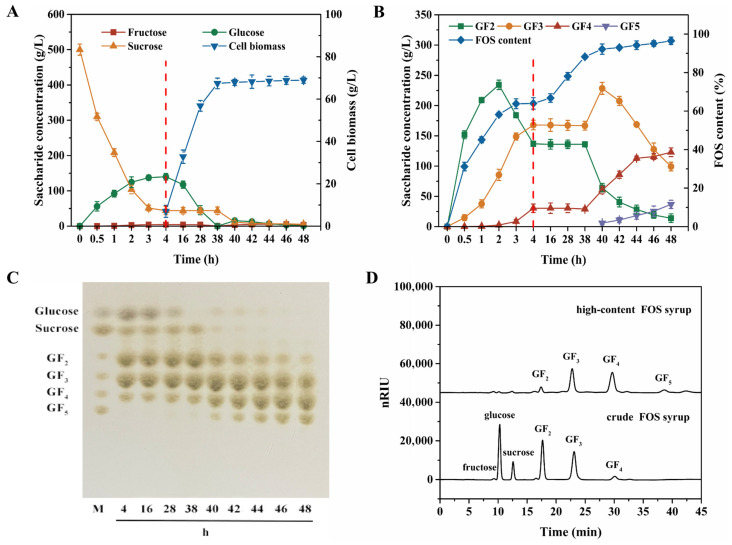
Time course of high-content FOS syrup production via sequential action of β-fructofuranosidase and *W. anomalus* CAU331. Concentrations of fructose, glucose, and sucrose (**A**). Concentration of GF_2_, GF_3_, GF_4_, GF_5_, and FOS content (**B**). TLC analysis (**C**). HPLC profiles of crude FOS syrup and high-content FOS syrup (**D**). The red dashed line demarcates the preparation stage of crude FOS syrup from the production stage of high-content FOS. Crude FOS syrup was first prepared by reacting 5 U/mL β-fructofuranosidase with 120 L of 500 g/L sucrose in a 200 L fermenter (45 °C, 100 rpm, 4 h), followed by heat inactivation at 100 °C for 5 min. The syrup was then fermented by *W. anomalus* CAU331 at 30 °C, 400 rpm, and 0.5 vvm aeration for 34 h. Subsequently, 5 U/mL β-fructofuranosidase was added and allowed to act synergistically with the yeast for an additional 10 h, while the pH was maintained at 6.0 by automated addition of NH_4_OH or H_3_PO_4_. All experiments were conducted in triplicate, and error bars indicate standard deviations.

A sequential strategy combining β-fructofuranosidase catalysis and yeast fermentation was developed to produce high-content FOS syrup from 500 g/L sucrose. Initially, a crude FOS syrup (64.0% FOS content) was obtained after 4 h in a 200 L fermenter (Figure 6A,B). The subsequent purification was conducted in two stages. During the initial stage (4~38 h), *W. anomalus* CAU331 was inoculated to purify the crude FOS syrup. The strain grew rapidly while consuming glucose, leading to a corresponding increase in FOS content. At 38 h, fructose and glucose were nearly depleted, raising the FOS content to 88.2%, while sucrose concentration remained at 44.0 g/L. In the second stage, β-fructofuranosidase was added at 38 h, resulting in a sharp decline in sucrose concentration. Fructose and glucose levels obviously increased, and both were subsequently metabolized by *W. anomalus* CAU331 (Figure 6A,C). Between 38 h and 48 h, GF_2_ content gradually decreased from 136.0 g/L to 13.9 g/L, while GF_3_ content significantly increased and then gradually reduced from 166.8 g/L to 99.3 g/L. In contrast, GF_4_ content was remarkably increased from 29.1 g/L to 122.9 g/L. GF_5_ was produced at 40 h and gradually increased from 5.9 g/L to 36.7 g/L due to transglycosidation (Figure 6B,C). Fructose, glucose, and sucrose concentrations were decreased to 3.4 g/L, 1.1 g/L, and 5.5 g/L, respectively (Figure 6A). This resulted in a FOS content of 95.1% (288.1 g/L total FOS), which further rose to 96.5% at 48 h. Compared with the crude FOS syrup, the high-content FOS syrup showed reduced peak areas for GF_2_ and GF_3_, with increased peak areas for GF_4_ and GF_5_ (Figure 6D).

Traditionally, the production of high-content FOS has employed strategies such as two-step fermentation [21], co-culture fermentation [22,34], or an immobilized FTase system [19]. However, these methods generally require a long reaction time and have low production efficiency, thus showing no distinct advantages for efficient production [19,21]. Therefore, a promising strategy that achieves high productivity, high product concentration, and a desirable FOS profile is highly valuable from an industrial perspective. In this study, a sequential action strategy based on β-fructofuranosidase and *W. anomalus* CAU331 was developed. The FOS content was up to 95.1% in a 200 L fermenter using 500 g/L sucrose as the substrate, and the FOS concentration was up to 288.1 g/L with productivity of 6.26 g/L/h, which is significantly higher than the results reported by the other studies (Table 1). Overall, the procedure in this study is more efficient and economically viable when compared to other strategies, which need longer process times and show lower productivity. For example, the one-step co-fermentation strategy was used for FOS production, and a high FOS content (93.8%) was achieved. However, a longer processing time (53 h) was required, which led to lower productivity [22]. Similarly, the immobilized FTase system required 72 h to reach 92.1% FOS content [19]. Though the FOS content achieved in this study is comparable to the recently reported mixed system (95.6%) [17], the present method has its own advantages, such as significantly higher productivity (6.26 g/L/h vs. 3.44 g/L/h), higher product concentration (288.1 g/L vs. 189.2 g/L), and the highest production of GF_5_ (25.5 g/L). Furthermore, high concentration of GF_4_ and GF_5_ in high-content FOS had better prebiotic effects and therefore improved application prospects and economic value [12]. The findings in this study established an economically efficient strategy for the production of high-content FOS, which exhibited great potential for industrial applications.

## 4. Conclusions

A novel yeast strain, *W. anomalus* CAU331, was isolated and identified for its ability to purify crude FOS syrup, and an economically efficient strategy was developed to produce high-content FOS. Through the sequential action of β-fructofuranosidase and *W. anomalus* CAU331 in a 200 L fermenter with 500 g/L sucrose as the substrate, a high-content FOS syrup of 95.1% was obtained at a productivity of 6.26 g/L/h. The total FOS concentration reached 288.1 g/L, comprising 19.2 g/L GF_2_, 127.8 g/L GF_3_, 115.8 g/L GF_4_, and 25.5 g/L GF_5_. Thus, the production process of high-content FOS syrup might be an excellent candidate strategy for application in industrial production.

## Figures and Tables

**Table 1:** Preparation of high-content FOS syrup from sucrose by different strategies.

Strain or Enzyme	Substrate	Strategy	Reaction Time (h)	FOS (g/L)	Productivity (g_FOS_/L/h)	Yield (g_FOS_/g_sucrose_)	FOS Content (%)	Reference
*A. pullulans* CCY27-1-94 and *S. cerevisiae* 11982	200 g/L sucrose	Two-step fermentation in 3 L fermenter	68	99.0	1.46	0.50	81.6	[21]
*A. ibericus* MUM 03.49 and *S. cerevisiae* YIL162W	200 g/L sucrose	Co-culture fermentation in 3.75 L fermenter	53	133.7	2.52	0.67	93.8	[22]
Fructosyltransferase (from *Penicillium brevicompactum*) and *B. coagulans*	600 g/L sucrose	Successive action of fed-batch fermentation and immobilized FTase in 1 L fermenter	72	128.6	1.79	0.21	92.1	[19]
β-fructofuranosidase (from *A. oryzae* S719) and *W. anomalus* GXL-22	300 g/L sucrose	Successive action of β-fructofuranosidase and yeast fermentation in 10 L fermenter	55	189.2	3.44	0.63	95.6	[17]
β-fructofuranosidase (from *A. niger* FBL-B) and *W. anomalus* CAU331	500 g/L sucrose	Sequential action of β-fructofuranosidase and yeast fermentation in 200 L fermenter	46	288.1	6.26	0.58	95.1	This study

## Data Availability

The original contributions presented in the study are included in the article, further inquiries can be directed to the corresponding author.

## Supplementary Materials

The following supporting information can be downloaded at: https://www.mdpi.com/article/10.3390/foods15030592/s1, Table S1. BBD experimental design matrix and results of FOS content by *W. anomalus* CAU331; Table S2. Analysis of variance (ANOVA) for the response surface quadratic model.
