# Supplementary material for: Fermentation by Wickerhamomyces anomalus Improved Production Yield of Fructooligosaccharides Through Transglycosidation of β-Fructofuranosidase
Source: Foods. 2026 Feb 6;15(3):592. doi: 10.3390/foods15030592 (PMC12897105; doi:10.3390/foods15030592)
Supplement: Supplementary file 1 [file foods-15-00592-s001.zip › Table S1.pdf]

Table S1. BBD experimental design matrix and results of FOS content by *W. anomalus* CAU331

Run	A- tryptone concentration (g/L)	B- initial pH	C- Inoculum size (%)	FOS content (%)
1	25(1)	5.5(-1)	0.3(0)	83.5±0.74
2	15(-1)	6.5(1)	0.3(0)	80.1±3.68
3	20(0)	6.0(0)	0.3(0)	87.6±2.89
4	20(0)	6.0(0)	0.3(0)	87.9±3.23
5	20(0)	5.5(-1)	0.1(-1)	65.7±2.09
6	20(0)	6.0(0)	0.3(0)	87.5±2.55
7	20(0)	6.0(0)	0.3(0)	86.8±1.24
8	15(-1)	5.5(-1)	0.3(0)	81.7±2.12
9	20(0)	6.5(1)	0.5(1)	83.3±1.12
10	15(-1)	6.0(0)	0.1(-1)	64.1±1.96
11	15(-1)	6.0(0)	0.5(1)	84.8±0.28
12	20(0)	5.5(-1)	0.5(1)	84.1±1.75
13	20(0)	6.5(1)	0.1(-1)	62.9±3.81
14	20(0)	6.0(0)	0.3(0)	87.1±3.46
15	25(1)	6.0(0)	0.1(-1)	70.3±1.56
16	25(1)	6.5(1)	0.3(0)	82.2±0.87
17	25(1)	6.0(0)	0.5(1)	85.5±1.49

Data are expressed as mean ± standard deviation (n=3).
