# Supplementary material for: Fermentation by Wickerhamomyces anomalus Improved Production Yield of Fructooligosaccharides Through Transglycosidation of β-Fructofuranosidase
Source: Foods. 2026 Feb 6;15(3):592. doi: 10.3390/foods15030592 (PMC12897105; doi:10.3390/foods15030592)
Supplement: Supplementary file 1 [file foods-15-00592-s001.zip › Table S2.pdf]

Table S2. Analysis of variance (ANOVA) for the response surface quadratic model

Source	Sum of squares	Degree of freedom	Mean square	F-value	*p*-value Prob > F
Model	1213.33	9	134.81	316.10	<0.0001
A	14.58	1	14.58	34.19	0.0006
B	5.28	1	5.28	12.38	0.0097
C	697.51	1	697.51	1635.43	<0.0001
AB	0.023	1	0.023	0.053	0.8249
AC	7.56	1	7.56	17.73	0.0040
BC	1.00	1	1.00	2.34	0.1696
A^2	11.67	1	11.67	27.37	0.0012
B^2	62.09	1	62.09	145.57	<0.0001
C^2	383.21	1	383.21	898.49	<0.0001
Residual	2.99	7	0.43		
Lack of Fit	2.24	3	0.75	3.99	0.1074
Pure Error	0.75	4	0.19		
Cor Total	1216.32	16			

R^2=0.9975; adjusted R^2=0.9944; adequate precision=47.975; CV=0.81
